# Emerging functions and clinical applications of exosomal microRNAs in diseases

**DOI:** 10.1016/j.ncrna.2023.05.004

**Published:** 2023-05-09

**Authors:** Soudeh Ghafouri-Fard, Hamed Shoorei, Peixin Dong, Yadollah Poornajaf, Bashdar Mahmud Hussen, Mohammad Taheri, Nader Akbari Dilmaghani

**Affiliations:** a Department of Medical Genetics, School of Medicine, Shahid Beheshti University of Medical Sciences, Tehran, Iran; b Clinical Research Development Unit of Tabriz Valiasr Hospital, Tabriz University of Medical Sciences, Tabriz, Iran; c Department of Anatomical Sciences, Faculty of Medicine, Birjand University of Medical Sciences, Birjand, Iran; d Department of Obstetrics and Gynecology, Hokkaido University School of Medicine, Hokkaido University, Sapporo, Japan; e Faculty of Medicine, Birjand University of Medical Sciences, Birjand, Iran; f Department of Clinical Analysis, College of Pharmacy, Hawler Medical University, Kurdistan Region, Erbil, Iraq; g Institute of Human Genetics, Jena University Hospital, Jena, Germany; h Urology and Nephrology Research Center, Shahid Beheshti University of Medical Sciences, Tehran, Iran; i Skull Base Research Center, Loghman Hakim Hospital, Shahid Beheshti University of Medical Sciences, Tehran, Iran

**Keywords:** Exosome, miRNA, Cancer, Neurological disorders, Cardiovascular disorders, Gastrointestinal disorders

## Abstract

Exosomes are an important group of extracellular vesicles that transfer several kinds of biomolecules and facilitate cell-cell communication. The content of exosomes, particularly the amounts of microRNA (miRNAs) inside these vesicles, demonstrates a disease-specific pattern reflecting pathogenic processes and may be employed as a diagnostic and prognostic marker. miRNAs may enter recipient cells through exosomes and generate a RISC complex that can cause degradation of the target mRNAs or block translation of their corresponding proteins. Therefore, exosome-derived miRNAs constitute an important mechanism of gene regulation in recipient cells. The miRNA content of exosomes can be used as an important tool in the detection of diverse disorders, particularly cancers. This research field has an important situation in cancer diagnosis. In addition, exosomal microRNAs offer a great deal of promise in the treatment of human disorders. However, there are still certain challenges to be resolved. The most important challenges are as follow: the detection of exosomal miRNAs should be standardized, exosomal miRNAs-associated studies should be conducted in large number of clinical samples, and experiment settings and detection criteria should be consistent across different labs. The goal of this article is to present an overview of the effects of exosome-derived microRNAs on a variety of diseases, including gastrointestinal, pulmonary, neurological, and cardiovascular diseases, with a particular emphasis on malignancies.

## Abbreviations

(**HUVECs**): Human Umbilical Vein Endothelial Cells
(**HUCMSCs**): Human Umbilical Cord Mesenchymal Stem Cells
(**hUCB-MSC**): Human Umbilical Cord Blood Mesenchymal Stem Cell
(**PBMCs**): Peripheral Blood Mononuclear Cells
(**BMMs**): Murine Bone Marrow Derived Monocytes and Macrophages
(**hCEp**): Human Cervical Epithelium Cell Line
(**hTERT-HPNE**): Normal Pancreatic Ductal Cell
(**hCC-MSCs**): Human Colon Cancer Mesenchymal Stem Cells
(**PSCs**): Pancreatic Stellate Cells
(**ucMSCs**): Umbilical Cord-Derived Mesenchymal Stem Cells
(**BMDMs**): Bone Marrow-Derived Monocytes
(**PSCs**): Primary Sertoli Cells
(**MDSCs**): Myeloid-Derived Suppressor Cells
(**USCs**): Urine-Derived Stem Cells
(**HPBCCs**): Human Primary Breast Cancer Cells
(**mTECs**): Mouse Tubular Epithelial Cells
(**WACs**): White Adipocytes
(**NSCs**): Neural Stem Cells
(**GSCs**): Glioma Stem Cells
(**HEMa-LP**): Human Epidermal Melanocytes
(**EPCs**): Endothelial Progenitor Cells
(**HCF**): Human Cardiac Fibroblast
(**ECs**): Endothelial Cells
(**CMECs**): Cardiac Microvascular Endothelial Cells
(**ILC2s**): Innate Lymphoid Cells
(**FNF**): Femoral Neck Fracture

## 1 Introduction

Exosomes are membrane-bound extracellular vesicles that are created in the endosomal section of the majority of eukaryotic cells. These vesicles can be detected in biofluids and have important function in physiological processes, particularly in intercellular communication. Being first identified as secreted particles from differentiated reticulocytes [1], exosomes were supposed to be only implicated in waste secretion from the cell for a long time. This changed when the importance of these vesicles in antigen presentation was discovered by Raposo et al. [2]. This observation threw light on the role of exosomes in the transmission of information between cells. In addition to participating in antigen presentation, exosomes have diverse roles in the regulation of immune response, modulation of several biological aspects of tumor cells such as migration and proliferation, and regulation of different types of cell death [3]. A new paradigm in this research area is the importance of exosomes in the transmission of miRNAs to attain intercellular communication [4]. These short-sized non-coding RNAs can go into the recipient cells using exosomes, and establish a RISC complex that can induce degradation of the target mRNAs or inhibit their translation into proteins. Thus, exosome-derived miRNAs constitute an important route of gene regulation in recipient cells. The importance of miRNAs in the pathobiology of human disorders has been reviewed in several studies [[5], [6], [7]]. The current review aims to provide an overview of the impact of exosome-derived miRNAs in diverse disorders, including gastrointestinal, pulmonary, neurological, and cardiovascular disorders, with a special focus on cancers.

## 2 Exosomal miRNAs in gastrointestinal disorders

**Fig. 1:**
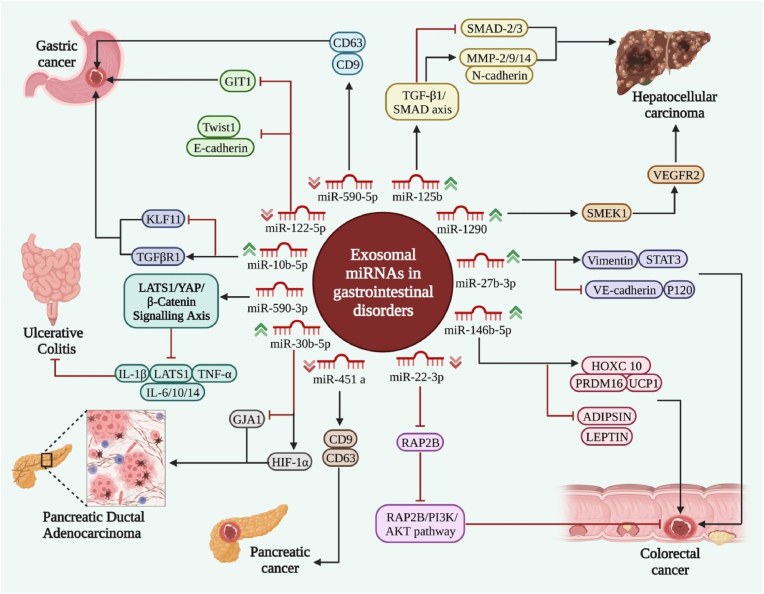
Exosomal miRNA signaling pathways in gastrointestinal disorders. Cancer cell-derived exosomal miRNAs play crucial roles in the prevention or promotion of gastrointestinal disorders through a variety of signaling pathways via influencing levels of targeted genes.

The impact of exosome-mediated delivery of miRNAs in the pathogenesis of gastrointestinal disorders has been assessed in malignant and non-malignant conditions (Table 1). In gastric cancer patients, serum exosomal levels of miR-590-5p have been lower in both early (stages I and II) and late (stage III) groups compared with healthy controls. The expression level of this miRNA could differentiate affected individuals from unaffected ones with an area under the receiver operating characteristic curve (AUC) value of 0.810. Most notably, serum levels of exosomal miR-590-5p have been related to clinical stage, infiltration depth, and levels of Ki-67. Moreover, authors have reported a correlation between the down-regulation of exosomal miR-590-5p and a low overall survival rate. In vitro studies have shown that over-expression of miR-590-5p leads to the suppression of cell migration and invasion in gastric cancer cells [8]. miR-122-5p is another downregulated miRNA in serum-derived exosomes of patients with this type of cancer. Exosome-mediated delivery of miR-122-5p could hamper the proliferation and metastatic ability of gastric cancer cells by inhibiting the expression of GIT1 [9]. Conversely, miR-10b-5p has been shown to be over-expressed in tissues and serum exosomes of advanced stages of gastric cancer compared with early-stage samples. Functional studies have shown that miR-10b-5p could target PTEN in gastric cancer cells and KLF11 in fibroblasts. miR-10b-5p silencing up-regulates PTEN levels and repression of PI3K/AKT/mTORC1 signals in gastric cancer cells, resulting in a reduction of colony formation ability and viability of these cells (Fig. 1). In fibroblasts, up-regulation of miR-10b-5p has resulted in down-regulation of KLF11 and elevation of TGFβR1 levels. Taken together, elevated exosomal levels of miR-10b-5p participate in the interactions between gastric cancer cells and fibroblasts in tumor niches by regulating TGF-β signals [10].

**Table 1:** Exosomal miRNAs in gastrointestinal disorders.

Type of Disease	miRNA/expression pattern	Human/Animal Samples	Cell Line	Targets & Pathways	Observation	Ref
Gastric Cancer (GC)	miR-590-5p (Down)	GC serum samples (n = 168) and healthy control serum samples (n = 50)	MGC-803, HGC-27	CD63, CD9	Exosomal miR-590-5p is considered as a diagnostic marker for GC.	[8]
GC	miR-122-5p (Down)	GC patient serum samples and healthy control serum samples	GES-1, AGS, MKN45, HGC27	GIT1, HSP70, TSG101, Twist1, E-cadherin	Exosomal miR-122-5p via down-regulating GIT1 could inhibit the tumorigenicity of GC.	[9]
GC	miR-10b-5p (Up)	GC tissue samples (n = 169) and healthy gastric mucosa samples (n = 27); GC patients' serum samples (n = 145) and healthy control serum samples (n = 178)	SGC-7901, MGC-803, 293T	PTEN, AKT, S6, TGFβR1, KLF11	Exosomal miR-10b-5p could mediate communication between GC cells and fibroblasts.	[10]
GC	miR-552-5p (Up)	BALB/c nude mice; GEO and TCGA databases	GES1, AGS, SGC-7901, MGC-803	PTEN, TOB1, Flag, E/N-cadherin, Bax, Vimentin, Bcl-2, Caspase-3	Exosomal miR-552-5p via regulating the PTEN/TOB1 axis could enhance tumorigenesis in GC.	[14]
Hepatocellular Carcinoma (HCC)	miR-638 (−)	male CB17.CgPrkdcscidLystbg-J/CrlCrlj mice	Huh-7-Luc, HUVECs	ZO-1, Snail, E/N-cadherin, VE-cadherin	Serum exosomal miR-638 via targeting ZO-1 and VE-cadherin could be considered as a prognostic marker of HCC.	[11]
HCC	miR-15a (−)	BALB/c nude mice	MSCs, SMMC-7721, Hep3B, Huh7	SALL4, TSG101, HLA-DR, PCNA, MMP-2/9, Caspase-3	Exosomal miR-15a from MSCs via down-regulating SALL4 could impede HCC progression.	[12]
HCC	miR-125b (Up)	HCC patients serum samples (n = 239) and non-HCC patient serum samples (n = 45)	SK-HEP1, SNU449, Huh7	HSP70, SMAD-2/3, MMP-2/9/14, E/N-cadherin	Exosomal miR-125b could enhance anti-metastatic and predict early metastasis of HCC.	[15]
HCC	miR-1290 (Up)	HCC and adjacent normal tissues (n = 49 pairs); HCC patient serum samples (n = 49) and healthy control serum samples (n = 28); BALB/c mice; NOD-SCID mice	HUVECs, L-02, Hep3B, HepG2, SMMC-7721, PLC/PRF/5	SMEK1, VEGFR2	Exosomal miR-1290 via Targeting SMEK1 could enhance the angiogenesis of HCC.	[16]
Colorectal Cancer (CRC)	miR-21-5p (Up)	Athymic BALB/c-nu/nu mice	HUVECs, 293A, LoVo, SW620, HT29, SW480, HCT116, LS174T	KRIT1, GM130, TSG101	Cancer-derived exosomal miR-21-5p could induce angiogenesis and vascular permeability.	[13]
CRC	miR-30a, miR-222 (−)	Nude mice	HCT-116, HT29, hCC-MSCs	MIA3, ALIX, HLA-DR	Exosomal miR-30a and miR-222 originated from CRC MSCs can enhance the tumorigenicity of CRC via targeting MIA3.	[17]
CRC	miR-146a-5p miR-155-5p (Up)	CRC and adjacent normal tissues (24 pairs), CRC metastasis and non-metastasis serum samples (n = 10 and n = 7, respectively), healthy control serum samples (n = 13); C57BL/6J mice; Athymic Balb/c-nu/nu mice; nude mice; TCGA databases	HCT116, SW620, MRC-5	CXCL12, CXCR7, E/N-cadherin, Vimentin, α-SMA, Snail, JAk2, STAT3, NF-κβ	Exosomal miR-146a-5p and miR-155-5p via crosstalk with CAFs could enhance CXCL12/CXCR7-associated metastasis.	[18]
CRC	miR-27b-3p (Up)	CRC tissue samples (n = 50) and non-tumor tissue samples (n = 50); CRC patient serum samples (n = 40) and healthy control serum samples (n = 10); BALB/c nude mice	HUVECs, LoVo, NCM460, SW480, HCT-116, DLD-1, SW620	VE-cadherin, Vimentin, HSC70, TSG101, STAT3	EMT-cancer cells-derived exosomal miR-27b-3p via modulating vascular permeability could enhance circulating tumor cells-mediated metastasis in CRC.	[19]
CRC	miR-193a, let-7g (−)	CRC patient serum samples (n = 69)	SNU-2335A/D, SNU-2404A/B, SNU-2414A/B, KM12C, KM1214, SW480, SW620	MMP-16, Snail, E-cadherin, ERK Vimentin	Exosomal miR-193a and let-7g could enhance cancer progression.	[20]
CRC	miR‐146b‐5p (Up)	C57BL/6 mice; CRC tissue samples (n = 48) and healthy control tissue samples (n = 48)	SV, 293T, HCT‐116, WACs	UCP1, PRDM16, LEPTIN, ADIPSIN, TSG101, CD81	CRC via transferring exosomal miR‐146b‐5p could increase adipose tissue browning and cancer cachexia.	[21]
CRC	miR-128-3p (−)	BALB/c nude mice; CRC patient serum samples (n = 66)	HCT-116, SW480	TGF-β, SMAD2/3, JAK2/3, STAT3, E/N-cadherin, Vimentin, ZO-1	Exosomal miR-128-3p via regulating the TGF-β/SMAD and JAK/STAT3 axis could enhance EMT in CRC cells.	[22]
CRC	miR-22-3p (Down)	36 pairs CRC and adjacent normal tissue samples	NCM460, HT-29, SW620, HCT-116, SW480, LoVo, hBMSCs	RAP2B, PI3K, AKT, CD9, CD63, HSP70	Exosomes miR-22-3p derived from MSCs via regulating RAP2B and the PI3K/AKT pathway can suppress CRC proliferation and invasion.	[23]
CRC	miR-10a (Down)	CRC patient serum samples (n = 40) and healthy control serum samples (n = 20); 15 pairs of CRC and adjacent normal tissues	NHLF, SW480	IL-6/8, IL-1β	Exosomal-miR-10a derived from CRC cells could decrease the migration of lung fibroblasts, and levels of IL-6, IL-8, and IL-1β.	[24]
Pancreatic Cancer (PC)	miR-451 (Down)	Serum samples (n = 191) and pancreatic benign disease patient serum samples (n = 95) and healthy control serum samples (n = 90)	–	CD9, CD63	Serum exosomal miR-451a could act as a marker for PC.	[25]
Pancreatic Ductal Adenocarcinoma (PDAC)	miR-30b-5p (Up)	PDAC patient tissue samples (n = 24) and healthy control tissue samples (n = 11); PDAC serum samples (n = 24) and healthy control serum samples (n = 11); C57BL6/J mice	AsPC-1, BxPC-3, MIA PaCa-2, PANC-1, T3M4, hTERT-HPNE, PaTu8988, 293T, HUVECs,	GJA1, TSG101, ALIX, HSP70, HIF-1α	Hypoxic PC-derived exosomal miR-30b-5p via inhibition of GJA1 expression could enhance tumor angiogenesis.	[26]
Chronic Pancreatitis (CP)	miR-130a-3p (Up)	SD rats	AR42J, PSCs	PPAR-γ, α-SMA, Coll-I/III,	Acinar cell-originated exosomal miR-130a-3p could regulate pancreatic fibrosis through affecting stellate cell PPAR-γ.	[27]
Ulcerative Colitis (UC)	miR-590-3p (−)	C57BL/6 mice	FHC, Thp-1,	LATS1, YAP, β-catenin, Caspase-3, TNF-α, IL-1β, IL-6/10/14, IL-12B	M2 macrophage-derived exosomal miR-590-3p by modulating the LATS1/YAP/β-catenin axis could enhance epithelial repair.	[28]
UC	miR-21a-5p (−)	UC tissue samples (n = 30) and healthy colon tissue samples (n = 30); C57BL/6 mice	PBMCs, FHC, THP-1	E-cadherin, ILC2, IL-4/5/6/11/13/22, TNF-α, GATA-3	M1 macrophage exosomes miR-21a-5p via ILC2 activation could aggravate UC.	[29]
–	miR-124 (−)	SD rats	BRL-3A, HUCB-MSCs	ALT, AST, Foxg1, chtf8	HUCB-MSC-originated exosomal miR-124 via down-regulating the Foxg1 could enhance rat liver regeneration following partial hepatectomy.	[30]

In hepatocellular carcinoma, exosomal levels of miR‐638 in serum samples have been shown to have a prognostic value through decreasing the expression of VE‐cadherin and ZO‐1 in endothelial cells [11]. On the other hand, the secretion of exosomal miR-15a from mesenchymal stem cells has been shown to impede the progression of this type of cancer through down-regulating SALL4 levels [12].

In colorectal cancer, several miRNAs have been discovered in cancer-derived exosomes that can affect the progression of this type of cancer. For instance, the exosome-mediated transmission of miR-21-5p from colorectal cancer cells to endothelial cells has repressed KRIT1 expression in recipient HUVEC cells and consequently induced activity of β-catenin signals and enhanced expression of VEGF-A and Ccnd1. Totally, these expression changes lead to the enhancement of angiogenesis and vascular permeability. Besides, levels of this miRNA in circulating exosomes have been shown to be elevated in patients with colorectal cancer compared with healthy donors [13]. In addition to the mentioned roles of miRNAs in the pathogenesis of malignant gastrointestinal disorders, exosome-derived miRNAs can affect the pathogenesis of chronic pancreatitis and ulcerative colitis (Table 1).

## 3 Exosomal miRNAs in lung cancer

**Fig. 2:**
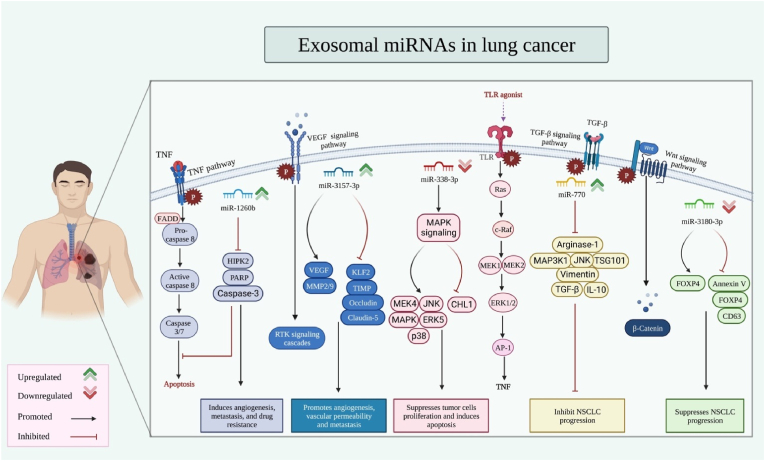
Exosomal miRNA signaling pathways. Exosomal microRNAs play crucial roles in the prevention or promotion of NSCLC through a variety of signaling pathways, depending on whether they are upregulated or downregulated.

Exosome-mediated delivery of miRNAs is also implicated in the pathogenesis of lung cancer (Table 2). For instance, lung cancer-derived exosomal miR-1260b has been shown to promote the metastatic ability of these cells via the suppression of HIPK2 expression [31]. Moreover, both angiogenic and metastatic abilities and vascular permeability can be enhanced by tumor-originated exosomal miR-3157-3p [32]. In addition, the secretion of miR-155 and miR-196a-5p in the exosomes of tumor-associated macrophages can enhance the metastasis of this type of cancer [33]. On the other hand, exosome-transferred miR-338-3p has a suppressive effect on the metastasis of lung cancer through the inhibition of CHL1 via the MAPK signaling pathway [34]. Exosome-mediated carriage of miR-770 suppresses M2 macrophage polarization by influencing the expression of MAP3K1. This would reduce the invasive abilities of lung cancer cells [35]. Similarly, exosomal miR‐3180‐3p can inhibit the proliferation and metastatic ability of lung cancer cells through the inhibition of FOXP4 expression [36] (Fig. 2).

**Table 2:** Exosomal miRNAs in lung cancer.

Type of Disease	miRNA/expression pattern	Human/Animal Samples	Cell Line	Targets & Pathways	Observation	Ref
Non-Small Cell Lung Carcinoma (NSCLC)	miR-1260b (Up)	124 pairs of NSCLC and adjacent normal tissues; NSCLC serum samples (n = 48) and healthy control serum samples (n = 48)	A549, Calu-1	HIPK2, PARP, Caspase-3	Exosomal miR-1260b derived NSCLC via inhibiting HIPK2 could promote tumor metastasis.	[31]
NSCLC	miR-3157-3p (Up)	Serum samples with and without metastasis (n = 50 pairs), healthy control serum samples (n = 50); 40 pairs of NSCLC and adjacent normal tissues; nude mice	H1299, SPCA1, PC9, A549, 16HBE, HUVECs	TIMP, KLF2, CD63, TSG101, VEGF, MMP-2/9, Occludin, ZO-1, Claudin-5	Tumor-derived exosomal miR-3157-3p via targeting the TIMP/KLF2 axis could enhance angiogenesis vascular permeability and metastasis in NSCLC.	[32]
NSCLC	miR-338-3p (Down)	TCGA, GEO databases; NSCLC serum samples (n = 7) and healthy control serum samples (n = 7)	BEAS-2B, A549, SK-MES-1	CHL1, MAPK, ERK5, JNK, MEK4, p38	Exosomal miR-338-3p via targeting the MAPK pathway by inhibiting CHL1 could suppress NSCLC cell metastasis.	[34]
NSCLC	miR-155, miR-196a-5p (−)	Metastatic and non-metastatic tissues (n = 15 pairs); Athymic BALB/c nude mice	A549, THP-1, A549/Luc	TSG101, TNF-α, IRF5, IRF4, Arg-1, E-cadherin, Vimentin, RASSF4	Exosomal miR-155 and miR-196a-5p could enhance the metastasis of NSCLC.	[33]
NSCLC	miR-770 (−)	TCGA databases; BALB/c nude mice	SK-MES-1, A549, NCI–H1650, BEAS-2B, THP-1 c	MAP3K1, TSG101, Arginase-1, iNOS, IL-10, TGF-β, E/N-cadherin, Vimentin, JNK, ERK1/2	Tumor cell-derived exosomal miR-770 via targeting the MAP3K1 by inhibiting macrophages could decrease invasion of NSCLC.	[35]
NSCLC	miR-3180-3p (Down)	GEO databases; nude mice	A549, HBE, BEAS-2B, PC9, H460, H226, H1299, H1703	FOXP4, Flotillin-1	Exosomal miR-3180-3p via downregulating FOXP4 could inhibit the proliferation and metastasis of NSCLC.	[36]

## 4 Exosomal miRNAs in breast and cervical cancers

Breast and cervical cancers are two types of cancers in which the role of exosomal miRNAs has been investigated (Table 3). Exosomes originating from cancer-associated fibroblasts (CAFs) have been shown to promote the proliferation and metastatic ability of breast cancer cells through transferring miR-500a-5p, a miRNA that inhibits the expression of USP28 [37]. Moreover, these exosomes transfer miR-18b to breast cancer cells to promote their invasion and metastasis via the regulation of TCEAL7 [38]. Meanwhile, the polarization of tumor-associated macrophages can be modulated by the miR-138-5p content of cancer-derived exosomes through the downregulation of KDM6B [39]. On the other hand, exosome-mediated transfer of miR‐134‐5p can confine the progression of breast cancer by regulating the PI3K/AKT pathway and by influencing the expression of ARHGAP1 [40].

**Table 3:** Exosomal miRNAs in female cancers.

Type of Disease	miRNA/expression pattern	Human/Animal Samples	Cell Line	Targets & Pathways	Observation	Ref
Breast Cancer (BC)	miR-500a-5p (−)	TCGA databases; BALB/c nude mice	MDA-MB-231, MCF7	USP28, α-SMA, FAP, FSP1, Vimentin, E/N-cadherin, FN1, ZEB1, Snail, Slug	Exosomal miR-500a-5p derived from CAFs via regulating USP28 could increase BC cell proliferation and metastasis.	[37]
BC	miR-138-5p (−)	BALB/c mice	MDA-MB-231, T47D, 4T1, THP-1, Raw 264.7	KDM6B, TNF-α, IL-6, IL-1β	Cancer-originated exosomal miR-138-5p via down-regulating KDM6B could regulate the polarization of tumor-associated macrophages.	[39]
BC	miR-145 (−)	Nude mice	MDA-MB-231, HUVECs	STIM1, IRS1, HSP70, c-Raf, ERK, p38, AKT, mTOR	STIM1 via reducing exosomal miR-145 could promote angiogenesis in MDA-MB-231 cells.	[43]
BC	miR-7641 (−)	BC patient serum samples (n = 28); BALB/c nude mice; TCGA databases	HPBCCs, MCF-7, MDA-MB-231, HCC-1937	CD9, CD63	Cancer-derived exosomal miR-7641 could enhance BC progression and metastasis.	[44]
BC	miR-18b (−)	Oncomine, GEO databases; BALB/c nude mice	MCF-7, MDA-MB-231	TCEAL7, α-SMA, β-Catenin, MMP-3/9, E/N-cadherin, Snail, Vimentin, Zeb1/2, Slug, ICAM-1	CAFs-derived exosomal miR-18b via regulating TCEAL7 could enhance BC invasion and metastasis.	[38]
BC	miR-134-5p (−)	23 pairs of BC and adjacent normal tissue samples; BALB/c nude mice	MCF-7, MCF-10A, MDA-MB-231,	ARHGAP1, TSG101, HSP70, Bcl-2, Bax, PI3K, AKT	Exosome miR-134-5p via regulating the PI3K/AKT pathway by targeting ARHGAP1 could restrain BC progression.	[40]
Cervical Cancer (CC)	miR-1468-5p (Up)	CC tissue samples (n = 102) and uterine leiomyoma patients tissue samples (n = 67); CC serum samples (n = 102) and healthy control serum samples (n = 67); B-NDG mice	Siha, Caski, HeLa, C33A, ME180, MS751, hCEp, HDLECs, PBMCs	PD-1, PD-L1, TSG101, HSP70, IFN-γ, HMBOX1, STAT3, SOCS1/2/3, JAK2	Cancer-secreted exosomal miR-1468-5p via immunosuppressive reprogramming of lymphatic vessels could accelerate tumor immune escape.	[41]
CC	miR-663b (Up)	59 pairs of CC and cervix tissue samples; Athymic nude mice	293T, SiHa, HeLa, CaSki, H8, HUVECs	Vinculin, TSG101, CD81, CD63	CC-derived exosomal miR-663b through inhibition of vinculin expression could increase angiogenesis in vascular endothelial cells.	[42]
CC	miR-125a-5p (Down)	CC patient serum samples (n = 44) and healthy control serum samples (n = 28)	–	–	Circulating exosomal miR-125a-5p could be considered a biomarker of CC.	[45]

In the context of cervical cancer, exosomes secreted by cancer cells contain miR-1468-5p that induces immune escape through the immunosuppressive reprogramming of lymphatic vessels [41]. Moreover, the delivery of miR-663b by these exosomes enhances the angiogenic ability of cervical cancer cells by inhibiting the expression of vinculin in vascular endothelial cells [42].

## 5 Exosomal miRNAs in brain disorders

Exosome-mediated transfer of miRNAs is also implicated in the pathogenesis of glioma, medulloblastoma, traumatic brain injury, neuroinflammation and Rett Syndrome (Table 4). For instance, miR-1246 has been found to be expressed in exosomes extracted from the body fluids of patients with glioma. This miRNA has an important role in the differentiation and activation of myeloid-derived suppressor cells. Exosomal levels of this miRNA in CSF samples after tumor resection have been associated with glioma recurrence rates. Moreover, the expression of miR-1246 in glioma-originated exosomes can be enhanced by hypoxia through a POU5F1 and hnRNPA1-dependent mechanism. Notably, the microtubule inhibitor 2-Methoxyestradiol has been shown to suppress the activation of myeloid-derived suppressor cells through the inhibition of hypoxia-induced exosomal miR-1246 expression [46]. Plasma levels of another exosomal miRNA, namely miR-2276-5p have been shown to have a potential diagnostic and prognostic role in this type of cancer [47]. Alternatively, the miR-944 content of exosomes originating from glioma stem cells has been shown to reduce the growth and angiogenic ability of glioma cells through the inhibition of AKT/ERK signaling [48]. Finally, the exosomal miR-21-5p content of urine-derived stem cells has been shown to enhance neurogenesis to reduce the progression of Rett syndrome through modulation of the EPHA4/TEK axis [49].

**Table 4:** Exosomal miRNAs in brain disorders.

Type of Disease	miRNA/expression pattern	Human/Animal Samples	Cell Line	Targets & Pathways	Observation	Ref
Glioma	miR-1246 (−)	CSF samples (n = 21) and GBM patient serum and CSF samples (n = 4) and healthy control serum and CSF samples (n = 4); nude mice	U87MG, U251, A172, PBMCs	IL-10, TGF-β, D-L1, STAT5, ERK, DUSP3, POU5F1, hnRNPA1, HIF-α	Exosomal miR-1246 from glioma patient body fluids could be involved in the differentiation and activation of MDSCs.	[46]
Glioma	miR-2276-5p (Down)	CGGA, TCGA, GEO, GEPIA1, and GEO2 databases; glioma patient serum samples (n = 124) and non-tumor glioma serum samples (n = 36)	LN229, U87	RAB13, CD9, CD63	Exosomal miR-2276-5p could be considered as a biomarker diagnostic for glioma.	[47]
Glioma	miR-944 (Down)	CGGA, TCGA databases; 5 pairs of glioma and adjacent normal tissues; BALB/c nude mice	HA1800, T98G, SHG44, U87MG, U251MG, HUVECs	AKT, ERK, VEGFC, Angiogenin-1, MMP-9/14, TSG101	GSCs-derived exosomal miR-944 via inhibiting the AKT/ERK signaling could reduce glioma growth and angiogenesis.	[48]
Medulloblastoma (MB)	miR-101-3p, miR-423-5p (Up)	Blood plasma samples	Daoy, D283 Med, THP-1, HMO6, 293T	EZH2, FOXP4	Exosomal miR-101-3p and miR-423-5p via targeting the FOXP4 and EZH2 could inhibit tumorigenesis in MB.	[50]
Traumatic Brain Injury (TBI)	miR-5121 (−)	C57BL/6; Thy1-GFP knock-in mice	BV2	RhoA-GTP, IL-1β, IL-6/10, iNOS, Arginase, ALIX	Released microglia exosomal miR-5121 could attenuate neurite outgrowth and synapse recovery of neurons after TBI.	[51]
Neuroinflammation (NI)	miR-409-3p (−)	C57BL/6 mice	P815, BV-2	Nr4a2, NF-κβ, TSG101, IL-1β, IL-6, TNF-α	Released-activated mast cells exosomal miR-409-3p via targeting the Nr4a2 by activating the NF-κB pathway could enhance microglial migration, activation, and NI.	[52]
Rett Syndrome	miR-21-5p (Down)	WT B6 mice	USCs, NSCs	EPha4, Tie2, TEK, CD9, CD63, CD81	Human USCs -derived exosomal miR-21-5p via targeting the EPha4/TEK axis and regulating neurogenesis could attenuate the progression of Rett syndrome.	[49]

## 6 Exosomal miRNAs in cardiovascular disorders

The impact of exosomal miRNAs in the pathogenesis of cardiovascular disorders has been investigated in the contexts of myocardial infarction, thrombosis, atherosclerosis and abdominal aortic aneurysm (Table 5). miR-143-3p content of exosomes from mesenchymal stem cells has been shown to protect against myocardial ischemia/reperfusion injury through the regulation of autophagy [53]. Moreover, endothelial progenitor cells have been shown to secrete miR-218-5p/miR-363-3p in their exosomes. These miRNAs can amend the pathogenic processes in the course of myocardial infarction through modulation of the p53/JMY signaling pathway [54]. Finally, dendritic cells-originated exosomal miR-494-3p has been found to promote angiogenesis after myocardial infarction [55].

**Table 5:** Exosomal miRNAs in cardiovascular disorders.

Type of Disease	miRNA/expression pattern	Human/Animal Samples	Cell Line	Targets & Pathways	Observation	Ref
Myocardial I/R Injury	miR-143-3p (−)	SD rats	MSCs, H9c2	LC3-I/II, CHK2, Beclin1	MSCs-derived exosomal miR-143-3p could suppress myocardial I/R injury via regulating autophagy.	[53]
Myocardial Infarction (MI)	miR-218-5p, miR-363-3p (−)	SD rats	EPCs, PMSCs, HCF	p53, JMY, α-SMA, CD31, Vimentin, VEGFR-2, Coll-I/III, Timp1/2/3/4	Exosomal miR-218-5p/miR-363-3p from endothelial progenitor cells could alleviate MI through affecting the p53/JMY axis.	[54]
MI	miR-494-3p (Up)	C57BL/6 mice; SD rats	BMDCs, CMECs, HL-1	VEGF, CD31	Dendritic cell-derived exosomal miR-494-3p could increase angiogenesis after MI.	[55]
MI	miR-328-3p (−)	BALB/C nude mice	H9C2	Caspase-3	MI cardiomyocytes-derived exosomal miR-328-3p via Caspase signaling could increase apoptosis.	[57]
MI	miR-152-5p, miR-3681-5p (Down)	STEMI patient serum samples (n = 10) and NSTEMI patient serum samples (n = 10) and negative control serum samples (n = 14)	–	–	Exosomal miR-152-5p and miR-3681-5p could be considered as possible markers for ST-segment elevation MI.	[58]
Thrombosis	miR-145, miR-885 (Down)	Covid-19 patient serum samples (n = 28) and non-Covid-19 serum samples (n = 10)	HUVECs	ACE2, TMPRSS2, Procaspase, Caspase-3,	Downregulation of miR-145 and miR-885 Could be considered a biomarker of thrombosis in Covid-19.	[56]
Arteriosclerosis	miR-501-3p (−)	Vascular patient serum samples (n = 51)	–	–	Up-regulation of exosomal miR-501-3p could contribute to the progression of vascular stiffness.	[59]
Atherosclerosis (AS)	miR‐512‐3p (−)	C57BL/6 mice	MSCs, ECs	Keap1, Caspase-3, TNF-α, IL-1β, IL-6, Bcl-2, Bax, Nrf2	Exosomal miR‐512‐3p derived MSCs via regulating the Keap1 could inhibit oxidized low‐density lipoprotein‐induced vascular endothelial cell dysfunction.	[60]
Abdominal Aortic Aneurysm (AAA)	miR-17-5p (Down)	C57BL/6 mice	ADSCs, Raw264.7	TXNIP, NLRP3, IL-1β, IL-18, Caspase-1, GSDMD-FL, GSDMD-N	Exosomal miR-17-5p from adipose-derived MSCs via suppressing the TXNIP-NLRP3 inflammasome could inhibit AAA.	[61]

Levels of exosomal miR-145 and miR-885 in serum samples from COVID-19 patients have been significantly correlated with D-Dimer levels. Notably, treatment of human endothelial cells with sera of COVID-19 patients has led to a reduction of miR-145 and miR-885 release, enhancement of apoptosis, and impairment in angiogenic properties. Cumulatively, exosomal miR-145 and miR-885 have been shown to participate in the modulation of thromboembolic events in the context of COVID-19 [56].

## 7 Exosomal miRNAs in bone disorders

Exosomal miRNAs have essential roles in the pathogenesis of osteosarcoma, bone metastasis, multiple myeloma, osteoarthritis, postmenopausal osteoporosis, and non-traumatic osteonecrosis of the femoral head (Table 6). Exosomal miR-21-5p originating from bone marrow mesenchymal stem cells has been shown to enhance the proliferation and invasion of osteosarcoma cells via targeting PIK3R1 [62]. Besides, exosomal miR-501-3p originating from osteosarcoma cells can enhance osteoclastogenesis and aggravate bone loss in these patients [63].

**Table 6:** Exosomal miRNAs in bone disorders.

Type of Diseases	miRNA/expression pattern	Human/Animal Samples	Cell Line	Targets & Pathways	Observation	Ref
Osteosarcoma (OS)	miR-21-5p (Up)	Oncomine databases; nude mice	U2OS, MG63, hFOB1.19, MSCs	PIK3R1/2, Bcl-2, Bax, AKT, MTOR	Exosomal miR-21-5p originated from bone marrow MSCs could enhance OS cell proliferation and invasion via targeting the PIK3R1.	[62]
OS	miR-501-3p (Up)	GEO databases; C57BL/6 mice	SJSA, hFOB, hBMDMs, MC3T3-E1, LM8, BMDMs, 293T	ALIX, HSP70, TSG101, Nfatc1, Acp5, PTEN, PI3K, AKT	OS-derived exosomal miR-501-3p could enhance osteoclastogenesis and aggravate bone loss.	[63]
Bone Metastasis (BM)	miR-19a (−)	GEO and TCGA databases; Athymic-nu/nu mice	MDA-MB-231, MCF7, T47D, RAW 264.7, BMMs	IBSP, PTEN, p65, AKT	Exosomal miR-19a and IBSP could stimulate osteolytic BM of ER-positive breast cancer.	[64]
Multiple Myeloma (MM)	miR-1305 (−)	Cohort study	RPMI 8226, THP-1	Nanog, Oct14, Sox2, GLUT1, HIF-2α, FGF2, IGF1, TGF-β, IL-10	Exosomal miR-1305 could be considered a biomarker of MM.	[65]
MM	miR-10a (−), miR-16 (Down)	GEO databases	–	EPHA8, IGF1R, CCND1	BMSCs derived exosomal miR-10a and miR-16 via targeting EPHA8 or IGF1R/CCND1 can participate in the progression of patients with MM.	[66]
Osteoarthritis (OA)	miR-206 (Down)	C57BL/6 mice	BMSCs	Elf3, Osteocalcin, ALP, TNF-α, IL-1β, IL-6	BMSCs-derived exosomal miR-206 via reducing the Elf3 could increase osteoblast proliferation and differentiation in OA.	[67]
OA	miR-125a-5p (Down)	Traumatic OA cartilage tissues (n = 30) and amputation cartilage tissues (n = 30); C57BL/6 mice	BMMSCs, 293T	E2F2	miR-125a-5p-abundant exosomes derived from MSCs could inhibit chondrocyte degeneration in traumatic OA via targeting E2F2.	[68]
Postmenopausal Osteoporosis (PMO)	miR-186 (−)	SD rats	hBMSCs, BMSCs	YAP, ALIX, CD81, CD63, BMP2, Mob1	Exosomal miR-186 derived from BMSCs via targeting the hippo pathway could enhance osteogenesis in PMO.	[69]
Non-traumatic Osteonecrosis of the Femoral Head (NONFH)	miR-100-5p (−)	NONFH patient tissue samples (n = 40) and FNF control group tissues (n = 40); SD rats	hBMSCs, 293T, HUVECs	BMPR2, SMAD1/5/9, PPARγ, ALIX, TSG101, Coll-I, RUNX2, Osteocalcin, VEGFA	Exosomal miR-100-5p through suppression of BMPR2/SMAD1/5/9 signaling can block osteogenesis of hBMSCs and angiogenesis of HUVECs.	[70]

Exosomal miRNAs also have an important role in the induction of bone metastasis in estrogen receptor (ER)-positive breast tumors. Exosomal levels of miR-19a and IBSP have been shown to be significantly elevated in bone-tropic ER + breast cancer cells, resulting in the over-expression of these transcripts in the circulation of patients. IBSP can assist in the transfer of exosomal miR-19a to osteoclasts to enhance osteoclastogenesis [64].

## 8 Exosomal miRNAs in other disorders

Exosomal miRNAs can also contribute to the pathogenesis of a variety of other malignant (Table 7) and non-malignant disorders (Table 8). Prostate cancer, oral squamous cell carcinoma, papillary thyroid carcinoma, melanoma, and diffuse large B-cell lymphoma are examples of the former types of disorders, while systemic lupus erythematosus, sepsis, diabetes, diabetic nephropathy, acute and chronic kidney injury, unilateral ureteral obstruction and varicocele are examples of the latter types of disorders.

**Table 7:** Exosomal miRNAs in other cancers.

Type of Diseases	miRNA/expression pattern	Human/Animal Samples	Cell Line	Targets & Pathways	Observation	Ref
Prostate Cancer (PC)	miR-183 (Up)	GEO, TCGA databases; Athymic NCr-nu/nu mice	RWPE-1, LNCaP, PC3	TPM1, CD63, HSP70	Suppression of cancer cell-derived exosomal miR-183 could suppress cell growth and metastasis in PC via influencing levels of TPM1.	[71]
Oral Squamous Cell Carcinoma (OSCC)	miR-130b-3p (Up)	OSCC and adjacent normal tissues (n = 20 pairs); nude C57BL/6 mice	OECM1, HUVECs	PTEN, TSG101, ALIX, CD63	Exosomal miR-130b-3p via targeting the PTEN could enhance progression and tubular formation in OSCC.	[72]
Papillary Thyroid Carcinoma (PTC)	miR-29a (Down)	PTC patient serum samples (n = 119) and healthy control serum samples (n = 100)	PTC-1, BCPAP, Nthy-ori3-1	TSG101	Serum exosomal miR-29a is a possible marker for PTC diagnosis and prognosis.	[75]
Melanoma (ML)	miR-106b-5p (Up)	ML and adjacent normal tissues (n = 36 pairs), primary ML serum samples (n = 24), metastasis ML serum samples (n = 12); TCGA, GEO databases; nude mice	A375, A2058, SK-MEL-1, SK-MEL-28, HEMa-LP	EphA4, E/N-cadherin, Fibronectin, Snail, PAN-Ago, ERK,	Exosomal miR-106b-5p derived from melanoma cells via targeting the EphA4 could enhance primary melanocytes EMT.	[76]
Diffuse Large B-Cell Lymphoma (DLBCL)	miR-107 (Down)	DLBCL patient serum samples (n = 42) and control group serum samples (n = 31); SCID mice; GEO, GEPIA databases	PBMCs, 293T, OCI-LY1, OCI-LY3, OCI-LY8	FOXO1, PEPCK, CCND1, P27, Bcl-2, CDK6, RRAGC	miR-107 could be considered a biomarker of DLBCL.	[77]

**Table 8:** Exosomal miRNAs in other non-malignant disorders.

Type of Diseases	miRNA/expression pattern	Human/Animal Samples	Cell Line	Targets & Pathways	Observation	Ref
Systemic Lupus Erythematosus (SLE)	miR-451a, (Down) miR-16 (−)	Active and inactive SLE patients (n = 42), healthy controls (n = 21), SLE patient serum samples	PBMCs	CD63, TSG101, TNF-α, IFN-γ, IL-6	Exosomal miR-451a could correlate with SLE and renal damage.	[73]
SLE	miR-146a (−)	Renal tissue samples (n = 41); SLE patient urinary samples (n = 41) and healthy control urinary samples (n = 20)	AB8/13	TSG101, Nucleoporin 62, GM-130, Aquaporin 1, TRAF6, TLR4, IRAK1	Urinary exosomal miR-146a could be considered as a marker of albuminuria, activity changes, and disease fares in SLE.	[74]
Sepsis	miR-1-3p (Up)	SD rats; sepsis patient serum samples (n = 3) and healthy control serum samples (n = 3)	HUVECs	SERP1, Caspase-3, Bax, Bcl-2, GRP78, IL-1β, iNOS, VEGF, MLC,	Sepsis plasma-derived exosomal miR-1-3p via targeting SERP1 could destruct endothelial cells.	[78]
Sepsis	miR-16-5p (Down)	C57BL/6 mice	ADSCs, RAW264.7	TLR4, TNF-α, IL-1β, IL-6/10, iNOS, Arg1,	Exosomal miR-16-5p from ADSCs could enhance TLR4-mediated M2 macrophage polarization in septic lung injury.	[79]
Diabetes	miR-29a, miR-29b, miR-29c (−)	T2D patient serum samples (n = 13) and healthy control serum samples (n = 8); C57BL/6J mice	MIN-6, C2C12, 3T3-L1, hepatocyte	Ago-2, CD63, CD9, TSG101, ALIX, AKT, p85α, GSK	β-cells of the pancreas via releasing the exosomal miR-29 family could control glucose homeostasis.	[80]
Diabetic Nephropathy (DN)	miR-let-7a (Down)	SD rats	BMSCs	USP22, Bcl-2, Bax, Caspase-3, GSH-Px, N-cadherin, Vimentin	BMSCs-derived exosomal miR-let-7a could play a protective role in DN via inhibiting USP22 expression.	[81]
Acute Kidney Injury (AKI)	miR-125b-5p (−)	–	HUCMSCs, HK-2, mTECs	p53, TSG101, ALIX, Cyclin-B1, Bcl-2, Caspase-3, Bax	Exosomal miR-125b-5p derived from MSCs could repair tubular kidneys via suppression of the p53 in ischemic AKI.	[82]
Chronic Kidney Disease (CKD)	miR-335-5p (−)	–	HK-2, RTEC	ADAM19, ASTN2, RCOR1, Coll-I/III, Vimentin, α-SMA, E/N-cadherin, IL-1β, TNF-α, IL-4/6/10	HUCMSCs-derived exosomal miR-335-5p via reducing the ADAM19 could attenuate inflammation and tubular epithelial–myofibroblast *trans*-differentiation of renal tubular epithelial cells.	[83]
Unilateral Ureteral Obstruction (UUO)	miR-21 (Up)	C57 mice	NRK-52E, NRK-49F	PTEN, TGF-β1, Rab27a, Fibronectin, α-SMA, PCNA, AKT	Exosomal miR-21 from tubular cells via targeting the PTEN and by activating fibroblasts could contribute to renal fibrosis in UUO.	[84]
Varicocele	miR-210-3p (Up)	Varicocele patient semen samples (n = 104); SD rats	PSCs	HSP70, CANX, Vimentin, α-SMA, inhibin-B	Seminal exosomal miR-210-3p could be considered as a possible indicator of Sertoli cell injury.	[85]
Skin Wound	let-7f-5p, miR-21-5p, miR-23a-3p, miR-125b-5p, miR-145-5p, let-7a-5p	BALB/C mice	ucMSCs, HUVECs, 293T	TP53INP1, HSP70, Caspase-3, Bax, Bcl-2	Hypoxic ucMSC-secreted exosomal miR-125b via targeting TP53INP1 could promote endothelial cell survival and migration in the process of wound healing.	[86]

Suppression of cancer cell-originated exosomal miR-183 has been suggested as an anti-cancer-modality for prostate cancer by affecting the expression of TPM1 [71]. Moreover, exosome-mediated transfer of miR-130b-3p has been shown to promote the progression of oral squamous cell carcinoma and tubular formation via affecting the expression of PTEN [72]. Two miRNAs, namely miR-451a [73] and miR-146a [74] have been shown to contribute to the pathogenesis of systemic lupus erythematosus. Serum levels of the former have been correlated with renal damage [73], and urinary exosomal levels of the latter have been considered as a marker of albuminuria, activity changes, and disease fares in this disorder [74].

## 9 Advances and challenges in clinical applications of exosomal microRNAs

Recent studies have shown that exosomal microRNAs serve as vital indicators and disease mediators owing to several characteristics [[88], [89], [90], [91]]. Exosomes are abundant in numerous bodily fluids and exosomal microRNAs can be collected with reasonable simplicity and little invasiveness [[88], [89], [90], [91]], making exosomal microRNAs as useful and feasible biomarkers for disease diagnosis. Because exosomal miRNAs are protected by a lipid bilayer, they are less likely to be degraded by the RNase than free miRNAs [92,93]. This property of exosomal miRNA enables the monitoring of changes in their expression during the course of a disease, as well as the manipulation of disease-related cell signaling in a longer-lasting way [88].

## 10 Diagnostic biomarker

Some particular exosomal miRNAs have high diagnostic usefulness in cancers, and their detection aids in early tumor identification. For instance, the signature based on these four microRNAs could successfully differentiate colorectal cancer samples from normal ones [94]. Moreover, serum exosomal miR-134 levels were considerably lower in patients with gastric cancer than in control subjects, and exosomal miR-134 correctly differentiated patients with gastric cancer from matched individuals [95]. In ovarian cancer, miR-1290 was overexpressed in serum exosomes and tissues relative to benign ovarian neoplasm; thus, it may serve as a biomarker for distinguishing ovarian cancer from benign disease [96].

## 11 Prognostic biomarker

In addition to their diagnostic value, exosomal miRNAs have been extensively studied in the prognosis of cancers [97]. Downregulation of serum exosomal miR-148a was associated to a worse clinical outcome in breast cancer patients. As a result, exosomal miR-148a in serum may be an important biomarker for breast cancer prognosis [98]. Patients with non-small cell lung cancer who had lower serum exosomal miR-382 levels had a poorer overall survival (OS) rate, suggesting that exosomal miR-382 seems to be an useful predictive biomarker for monitoring the course of non-small cell lung cancer [99]. Plasma exosomal miR-130a levels were significantly higher in 184 patients with oral squamous cell carcinoma than in 196 healthy controls [100]. It was discovered that exosomal miR-130a is an independent predictor of overall survival and recurrence-free survival [100]. Thus, exosomal miR-130a has the potential to serve as a prognostic biomarker in the treatment of oral squamous cell carcinoma. Analysis of 125 patients with colorectal cancer, 70 healthy controls, and 45 benign adenomas revealed that serum exosomal miR-874 was significantly downregulated in colorectal cancer patients [101]. Low serum levels Expression of exosomal miR-874 was related to distant metastatic positivity, lymph node metastasis positivity, poor differentiation, advanced TNM stage, and worse survival [101]. Therefore, exosomal miR-874 serum expression may serve as a reliable colon cancer prognostic marker.

## 12 Therapeutic target

Exosomal microRNAs are rapidly being explored as a possible technique for treating cancers. On the basis that exosomal miRNAs effectively attach to target mRNA and decrease gene expression in recipient cells, malignancies have been treated with tumor suppressor exosomal miRNAs utilizing exosomal engineering techniques [89]. By blocking the MNK/eIF4E axis, exosome-mediated transportation of miR-7-5p improves the anti-cancer effect of Everolimus in non-small cell lung cancer [102]. In addition, exosomes carrying miR-34a promote apoptosis and restrict the migration and development of colorectal cancer cells [103]. Exosomal miR-499 inhibited tumor formation and angiogenesis [104] in endometrial cancer patients, where miR-499 expression was significantly decreased in cancer tissues compared to surrounding tissues. Exosomes that co-deliver 5-fluorouracil and miR-21 inhibitors are able to overcome the 5-fluorouracil (FU) resistance of colon cancer cells and significantly boost their toxicity [105]. miR-34c is a tumor suppressor miRNA that diminishes both malignant behavior and radioresistance in nasopharyngeal cancer [106]. Moreover, exosomes that overexpress miR-34c inhibit tumor formation and enhance the effectiveness of radiation treatment [106].

## 13 Challenges in clinical applications of exosomal microRNAs

Exosomal miRNAs offer a great deal of promise, however, there are still certain challenges to be resolved. For example, the detection of exosomal miRNAs should be standardized [107]. Until now, most exosomal miRNAs-associated studies have only been conducted with just a small number of clinical samples used, and experiment settings and detection criteria vary from lab to lab [107]. Additionally, no standardized techniques exist for collecting exosomes, deconstructing them, extracting, and storing miRNA [107]. Furthermore, the vast majority of investigations focused on exosomal miRNA levels in serum and plasma. In contrast, exosomes can be found in a range of physiological fluids, including saliva, tears, and urine [108]. Therefore, before exosomal miRNAs may be utilized as a diagnostic tool in clinical testing, more research and testing will be required to broaden liquid biopsy to uncommon sample sources.

The clinical use of exosomal miRNAs in future cancer treatment has faced some challenges [109]. The effectiveness of exosome-mediated treatment largely relies on exosome source, loading technique, and cell uptake [110]. The first difficulty is producing enough exosomes for clinical trials on a large scale. Bioreactors, 3D scaffolds, and microfluidic devices are used to increase the amount of exosomes [110]. However and quality should be assured while output is going up, especially since exosomes and other kinds of extracellular vesicles can be contaminated or have the same size [110]. The second difficulty is to discover new innovative ways for effectively loading nucleic acids into exosomes, since the poor loading efficiency of existing exosome-nucleic acid loading procedures limits their use [111]. The third challenge is to create precise cancer therapies that are personalized to each individual. The variety of exosomes and the complexities of the *in vivo* environment make it difficult to predict how and how successfully exosomal miRNA-based therapies will function [112].

## 14 Conclusions

Exosome-mediated delivery of miRNAs is implicated in the pathogenesis of a wide array of human disorders. Moreover, the miRNA content of exosomes can be used as an important tool in the detection of diverse disorders, particularly cancers. This research field has an important situation in cancer diagnosis, since cancer cells have been found to secrete higher quantities of exosomes compared with normal cells, and cancer-originated exosomes have a crucial impact on intercellular communications via transporting a variety of growth factors, chemokines, and miRNAs [87], the latter being the focus of this review. The data presented above indicate distinctive expression profiles of miRNAs in the exosomes originating from distinct cellular origins. This finding further highlights the possible application of these vesicles in diagnostic and prognostic approaches. miRNA-loaded exosomes are released not only from cancer cells but also from various immune and mesenchymal cells in the tumor niche, thus they exert diverse roles in cancer biology and affect different aspects of tumor progression, including proliferation, migration, and metabolic states of cancer cells. The content of these exosomes may also affect the response of patients to diverse therapeutic options, including both conventional and targeted therapies. Therefore, these vesicles can be used for establishing personalized routes of cancer treatment. In addition to malignant conditions, exosomal miRNAs affect the pathogenesis of a variety of non-malignant disorders, including immune-related ones such as systemic lupus erythematosus, degenerative disorders, and neurological disorders. Notably, exosomes contain several compounds that may have synergistic effects on recipient cells. Thus, a comprehensive evaluation of exosome cargo is required to determine the precise mechanisms behind their cellular influence. This research field would benefit from the application of novel strategies for exosome isolation and high-throughput sequencing methods for the identification of several targets which are affected by these vesicles.

## Ethics approval and consent to participant

Not applicable.

## Consent of publication

Not applicable.

## Availability of data and materials

The analyzed data sets generated during the study are available from the corresponding author on reasonable request.

## Authors’ contributions

SGF and PD wrote the manuscript and revised it. MT supervised and designed the study. HS, NAD, YP and BMH collected the data and designed the figures and tables. All authors read and approved the submitted version.

## Declaration of competing interest

The authors declare they have no conflict of interest.

Not applicable.
